# Analysis of Binding Modes between Three Perfluorosulfonates and GPER Based on Computational Simulation and Multiple Spectral Methods

**DOI:** 10.3390/toxics12050315

**Published:** 2024-04-26

**Authors:** Wenhui Liang, Yanting Chen, Yuchen Wei, Zeyu Song, Cancan Li, Yanhong Zheng, Zhongsheng Yi

**Affiliations:** College of Chemistry and Bioengineering, Guilin University of Technology, Guilin 541004, China; liang_lewihor@foxmail.com (W.L.); tax@glut.edu.cn (Y.C.);

**Keywords:** G protein-coupled estrogen receptor, perfluorochemical, spectroscopy, molecular dynamics simulation, molecular docking

## Abstract

Perfluorinated compounds (PFCs) belong to a significant category of global environmental pollutants. Investigating the toxicological effects of PFCs within biological systems is of critical significance in various disciplines such as life sciences, environmental science, chemistry, and ecotoxicology. In this study, under simulated human physiological conditions (pH = 7.4), a combination of multiple spectroscopic techniques and computational simulations was employed to investigate the impact of perfluorinated compounds (PFCs) on the G protein-coupled estrogen receptor (GPER). Additionally, the research focused on exploring the binding modes and toxicological mechanisms between PFCs and GPER at the molecular level. All three perfluorinated sulfonic acids (PFSAs) can induce quenching of GPER fluorescence through static quenching and non-radiative energy transfer. Steady-state fluorescence calculations at different temperatures revealed apparent binding constants in the order of 10^6^, confirming a strong binding affinity between the three PFSAs and GPER. Molecular docking studies indicated that the binding sites of PFSAs are located within the largest hydrophobic cavity in the head region of GPER, where they can engage in hydrogen bonding and hydrophobic interactions with amino acid residues within the cavity. Fourier transform infrared spectroscopy, three-dimensional fluorescence, and molecular dynamics simulations collectively indicate that proteins become more stable upon binding with small molecules. There is an overall increase in hydrophobicity, and alterations in the secondary structure of the protein are observed. This study deepens the comprehension of the effects of PFCs on the endocrine system, aiding in evaluating their potential impact on human health. It provides a basis for policy-making and environmental management while also offering insights for developing new pollution monitoring methods and drug therapies.

## 1. Introduction

Perfluoroalkyl sulfonic acid (PFSAs) is widely recognized as one of the most significant chemical products of the 20th century. Fluoride usage is prevalent in both the production and consumption of the organic industry [1,2]. PFSAs find extensive application in various domains, including surface antifouling agents, foam fire extinguishing agents, paint additives, pesticides, and herbicides, because of their desirable oil and water-repellent properties. Due to their strong persistence, toxicity, and bioaccumulation, PFSAs pose a significant challenge in terms of organic pollutant decomposition in nature [3,4,5]. Recent research indicates that PFSAs have emerged as a new type of environmental pollutant [6,7]. PFSAs tend to accumulate in organisms at higher levels compared to organochlorine pesticides and dioxins. PFSAs are capable of entering the human body through various pathways, such as food consumption, drinking water intake, and inhalation, and tend to amass within vital organs like the circulatory system, liver, kidneys, and brain, making them difficult to metabolize and eliminate from the body. Research has revealed that PFSAs exhibit various biological toxicities, including genotoxicity, reproductive toxicity, neurotoxicity, and developmental toxicity, among others. These toxic effects can directly or indirectly impair genetic material, leading to a range of diseases and even the formation of cancerous tumors [8]. In May 2009, the Stockholm Convention on persistent organic pollutants officially included perfluorooctyl sulfonic acid in the blacklist of persistent organic pollutants [9,10,11].

The G protein-coupled estrogen receptor (GPER) is an important protein in the human body and belongs to G proteins [12]. GPER is widely distributed in various organs of the human body, including the ovary, mammary gland, placenta, and uterus. This protein exhibits high affinity and acts as the target for a variety of drugs [13,14]. While traditional theory suggests that GPER ligands bind to extracellular sites, recent studies have demonstrated that when the ligands are fat-soluble substances, GPER and ligands can also interact in the cytoplasm or nucleus, resulting in functional effects [15,16]. PFSAs exhibit profound reproductive and developmental toxicity in organisms, leading to the occurrence of deformities and mortality among offspring. Therefore, it is particularly important to explore the combination mechanism and mode of action between GPER and PFSAs. Studying the combination mechanism of GPER and PFSAs can better predict and assess the biological activity and environmental behavior of these substitutes, which is of significant importance for developing new therapeutic strategies and drug designs.

This paper combines fluorescence spectra, UV–Vis spectra, infrared spectra, and computer simulation methods, such as molecular docking and molecular dynamics simulation. This study delved into the intricate mechanisms by which potassium perfluorobutyl sulfonate (PFBS), potassium perfluorohexyl sulfonate (PHFS), and potassium perfluorooctyl sulfonate (PFOS) affect the GPER receptor under controlled experimental conditions that simulate the human environment.

## 2. Experimental Materials and Methods

### 2.1. Materials and Reagents

Reagent: G protein-coupled estrogen receptor (GPER, 400 uL, Peptide affinity purified, Biorbyte, Cambridge, UK); Tirs HCl with pH = 7.4 was prepared at a concentration of 1.0 × 10^−7^ mol·L^−1^ and stored in the refrigerator for future use. Potassium perfluorobutylsulfonate (PFBS), potassium perfluorohexyl sulfonate (PFHS), and potassium perfluorooctyl sulfonate (PFOS) were obtained from Shanghai Maclin Biochemical (Shanghai, China) with a purity of ≥ 95%. Tris-HCl buffer solution (pH = 7.4) was prepared in the laboratory.

### 2.2. In Silico Methods

All computational simulations in this paper were completed on the Dell RedHat Linux 6.4 (Raleigh, NC, USA) operating system. The PFSAs small molecule was optimized at the B3LYP/6-31G* theoretical level using the density functional method implemented in Gaussian 16 software. The obtained conformation of the small molecules was utilized for molecular docking and molecular dynamics simulation. Protein crystal structures are derived from the sequence data of commercial proteins and are utilized to construct protein structures through homology modeling on specialized protein structure websites. https://zhanglab.ccmb.med.umich.edu/ (accessed on 24 March 2018) [17]. The crystal structure with the lowest energy was selected for the entire simulation process.

Molecular docking: The crystal structure of the protein and small molecules obtained previously were used for molecular docking. Autodock software 1.5.7 (The Scripps Research Institute, La Jolla, CA, USA) [18] was utilized to perform basic processing on the GPER protein. Phe208 was selected as the flexible residue for docking. The docking grid was set to dimensions of 60 Å × 60 Å × 60 Å with a grid spacing of 0.375 Å for site-specific docking (cavity). To obtain multiple conformations, the Lamarckian genetic algorithm [19] was utilized, simulating 20 different conformations over 25,000,000 steps.

Molecular dynamics simulation: Firstly, the small molecule ligands optimized by Gaussian 16 software were converted to the required file format through an online conversion website http://davapc1.bioch.dundee.ac.uk/cgi-bin/prodrg (Accessed on 24 March 2018) [20]. The simulated force field was set to gromos96 43a1. The genbox tool was used to add water molecules with the SPC model to the system, along with five chloride ions as counterions to maintain system neutrality. Initial energy minimization was performed using the steepest descent method until the total system energy was below 1000 kJ·mol^−1^·nm^−1^. The system was then equilibrated using the canonical ensemble (NVT) and the isothermal–isobaric ensemble (NPT), followed by a 20 ns molecular dynamics simulation. The molecular dynamics simulation calculations were conducted using Gromacs version 4.6.5.

In this study, temperature coupling is achieved using the modified Berendsen thermostat with the V-rescale method. To ensure more accurate control, two coupling groups are employed: one for the protein and another for non-protein components. The time constant (tau_t) for both groups is set to 0.1 ps. Additionally, a reference temperature of 300 K is assigned to each group. Electrostatic interactions are managed using the Particle Mesh Ewald (PME) method for long-range interactions, employing fourth-order cubic interpolation with a Fourier grid spacing of 0.16 nm.

### 2.3. Experimental Methods

Fluorescence spectrum data processing: To initiate the process, accurately transfer 1 mL of 1.0 × 10^−6^ mol·L^−1^ GPER solution into a 10 mL colorimetric tube. Then, add PFBs, PFHs, and PFOS with a concentration of 1.0 × 10^−5^ mol·L^−1^, followed by Tris HCl buffer solution to dilute to the scale mark. The reactions were conducted at temperatures of 293 K, 298 K, and 303 K for a duration of 10–15 min, with an excitation wavelength set at 280 nm and an emission wavelength of 5 nm. Both excitation and emission slits were maintained at 5 nm, and the scanning speed was set to 1200 nm·min^−1^, with a rated voltage of 700 V. By utilizing Tris-HCl buffer solution as the reference solution, the corresponding fluorescence spectrum of the measured object was measured.

Three-dimensional fluorescence spectra: At a constant temperature of 289 K, a solution containing 100 μL of PFSA small molecules at a concentration of 1.0 × 10^−5^ mol·L^−1^ was added into a 10 mL GPER colorimetric tube, which originally had a concentration of 1.0 × 10^−7^ mol·L^−1^. The reaction was maintained for a duration of 10–15 min. The instrument was configured with excitation and emission wavelengths ranging from 200 to 450 nm with a 5.0 nm increment. The slit width and scanning speed were both set to 5.0 nm and 1200 nm·min^−1^, respectively. The response time was set to 2.0 s. Three-dimensional fluorescence spectra were separately acquired for free GPER and three different PFSA–GPER complex systems.

UV–Vis absorption spectrum: The preparation concentration of the PFSAs small molecule is 1.0 × 10^−5^ mol·L^−1^. Absorbance measurements were conducted at room temperature using a Tris-HCl buffered solution as the reference blank solution. The measurements were performed individually for three types of PFSA compounds, and wavelengths ranging from 300 to 450 nm were used.

Fourier transform infrared spectroscopy (FTIR): KBr was ground and subsequently pressed into tablets using a tablet press. A Tris-HCl buffered solution was used to establish a blank background, which was then subtracted. Using a pipette, 10 μL of the sample was precisely dispensed onto the KBr window. The scanning parameters included a range from 4000 to 400 cm^−1^, a resolution of 4 cm^−1^, and two scanning repetitions. The infrared spectra of both free GPER and GPER-PFCA complex systems were determined separately. Following the experiment, the infrared spectral data within the wavelength range of 1600–1700 cm^−1^ were used for fitting and analyzing the changes in the protein’s secondary structure before and after binding.

## 3. Mechanism Discussion

### 3.1. Unraveling the Dynamics and Binding Affinity in the Interaction between GPER and PFSAs by Steady-State Fluorescence Spectrum

**Figure 1:**
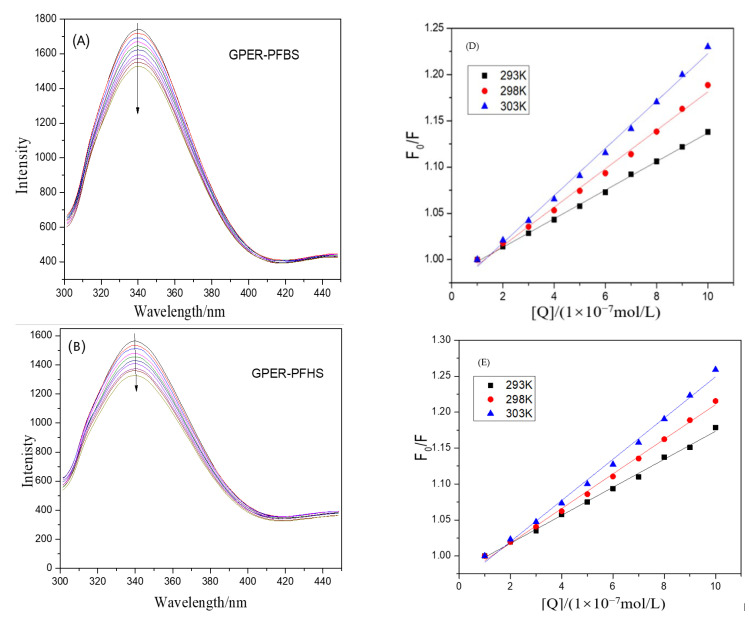

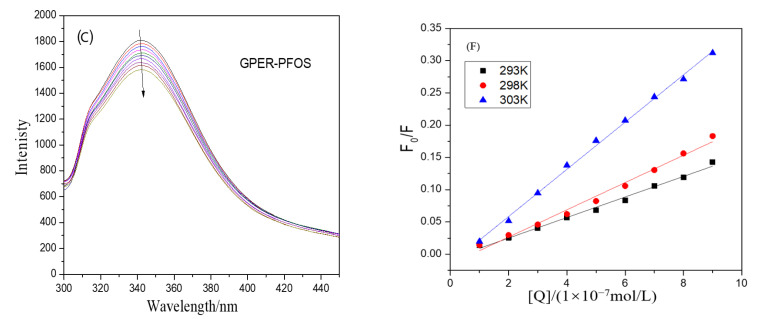
The fluorescence quenching spectrum of GPER at different concentrations of PFBS (**A**), PFHS (**B**), and PFOS (**C**): *C*_GPER_ = 1 × 10^−7^ mol·L^−1^, *C*_PFSAs_ = 0,1,2,3,4,5,6,7,8,9 (×10^−7^ mol·L^−1^), The direction of the arrows indicates that the concentration of PFSAs is gradually increasing. And the *F*_0_*/F* of PFSA–GPER interactions at different temperatures, PFBS–GPER (**D**), PFHS–GPER (**E**), and PFOS–GPER (**F**).

The fluorescence quenching of proteins can be primarily categorized into three mechanisms: static quenching, dynamic quenching, and non-radiative energy transfer [21]. Figure 1 depicts the fluorescence quenching spectra of GPER induced by PFSAs. The Stern-Volmer equation [22,23,24] was employed to calculate and analyze the obtained data.

(1) $$F_{0}/F=1+K_{\mathrm{sv}}\left[Q\right]=1+K_{q}\tau_{0}\left[Q\right]$$

(2) $$\log\left(F_{0}-F\right)/F=logK_{a}+nlog\left[Q\right]$$

In the equation, *F*_0_ and *F* represent the fluorescence intensities of the free GPER and GPER–PFSA complex, respectively. *K_sv_* is the fluorescence quenching rate constant, [*Q*] is the concentration of the ligand small molecule, *K_q_* is the bimolecular quenching rate constant, *τ*_0_ is the intrinsic fluorescence lifetime of the protein molecule before quenching, which is approximately 10^−8^ s, *K_a_* is the apparent binding constant, and *n* is the number of binding sites. As shown in the figure, we can observe that the strongest fluorescence peak appears at a wavelength of about 340 nm. The endogenous fluorescence waveform of GPER remains unaltered upon the addition of PFSAs, and the fluorescence intensity is consistently quenched. Figure 1B,C show a redshift of approximately 2 nm and 5 nm during the quenching process. These observations suggest that the binding of the small-molecule ligand to the amino acid residues within the cavity of GPER alters the protein’s native microenvironment, leading to the quenching of intrinsic fluorescence. Table A1 presents the various constants related to the interaction between GPER and PFSAs, as calculated using the formula. The obtained data reveal that the *K_q_* values are consistently higher than the maximum dynamic quenching constant of 2 × 10^10^, indicating that the fluorescence quenching is attributed to static quenching. Furthermore, the *K_a_* value falls within the range of 10^6^, signifying a strong binding affinity between the small-molecule ligand and the protein macromolecule. The binding site number (n) is approximately equal to 1, suggesting the presence of only one binding site between them.

### 3.2. Mechanisms and Analysis of Fluorescence Quenching by PFSAs: Static Quenching and Non-Radiative Energy Transfer in GPER

The mechanisms of fluorescence quenching primarily encompass static quenching, dynamic quenching, and non-radiative energy transfer [25]. The aforementioned findings suggest that the quenching of GPER by PFSAs is predominantly attributed to static quenching. Subsequently, further evaluation will be conducted to determine whether the quenching process involves non-radiative energy transfer through Förster dipole–dipole energy transfer theory. The fluorescence spectra of the protein should be superimposed with the ultraviolet–visible absorption spectra of PFSAs, followed by the utilization of Equations (3)–(5) [26,27] to calculate and analyze the obtained results.

(3) $$J=\sum F\left(\lambda \right)\varepsilon (\lambda )\lambda^{4}\Delta \lambda /\sum F(\lambda )\Delta \lambda$$

(4) $$R_{0}^{6}=8.8\times 10^{-25}K^{2}N^{-4}\phi J$$

(5) $$E=1-F/F_{0}=R_{0}^{6}/\left(R_{0}^{6}+r^{6}\right)$$

In the formula, *F*_0_ signifies the fluorescence intensity of unbound GPER, while *F* denotes the fluorescence intensity of the protein at a molar ratio of 1:1 between GPER and PFCAs. *J* represents the overlap integral of the fluorescence emission spectrum and the ultraviolet absorption spectrum, and *R*_0_ is the critical transfer distance at *E* = 50%. *r* signifies the binding distance between the energy ligand and the receptor. *K*^2^ assumes a value of 2/3, while *N* represents the refractive index of the medium, which has an average value of approximately 1.34 for water and organic compounds. *Φ* denotes the fluorescence quantum yield of GPER protein, which is approximately 0.15, while *E* represents the energy transfer efficiency of the entire complex system.

**Figure 2:**
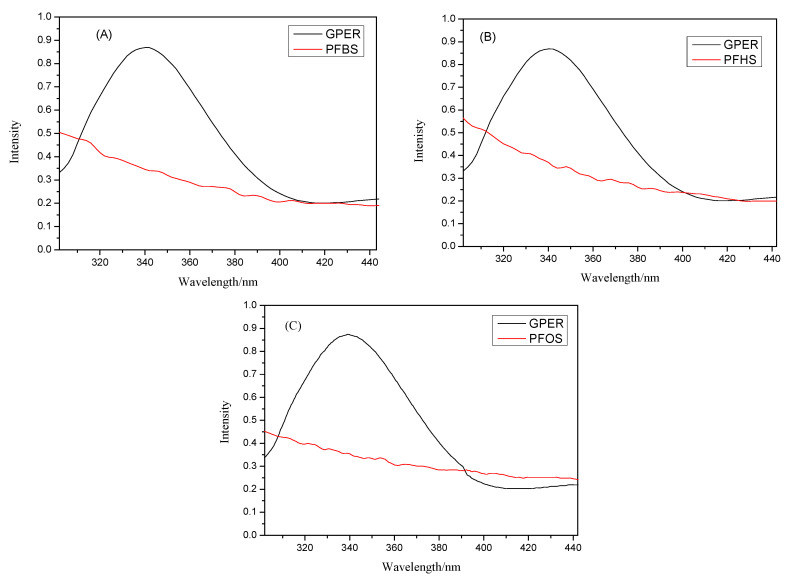
The overlap of the fluorescence spectrum of GPER and the UV absorption spectra of PFBS (**A**), PFHS (**B**), and PFOS (**C**). C_GPER_ = 1.0 × 10^−7^, C_PFSAs_ = 1.0 × 10^−7^.

As shown in Figure 2, the ultraviolet–visible absorption spectra of three PFSA small molecules overlap with the fluorescence spectra of GPER. The experimental data should be processed and analyzed using Formulas (3)–(5) provided earlier. After computation, the spectral overlap integral *J*, critical transfer distance *R*_0_, energy transfer efficiency *E*, and binding distance *r* between PFBS and GPER were determined to be *J* = 6.70 × 10^−14^, *R*_0_ = 3.50 nm, *E* = 0.13, and *r* = 4.87 nm, respectively. After analysis, it was found that the value of *r* falls within the range of 0.5 *R*_0_ to 1.5 *R*_0_ and is less than 7 nm, satisfying the conditions for energy transfer as per reference [28]. An analysis of the data for GPER and PFHS reveals the following values: *J* = 5.98 × 10^−14^, *R*_0_ = 3.44 nm, *E* = 0.18, *r* = 4.43 nm. Similarly, these results fulfill the conditions for non-radiative energy transfer. The experimental data analysis for GPER and PFOS reveals: *J* = 1.63 × 10^−14^, *R*_0_ = 4.07 nm, *E* = 0.21, *r* = 5.07 nm. These results indicate compliance with the conditions for non-radiative energy transfer. Similarly, it can be demonstrated that non-radiative energy transfer also occurs between GPER and PFOS. In summary, the introduction of small-molecule PFSAs as pollutants causes fluorescence quenching in GPER due to the combined effects of static quenching and non-radiative energy transfer.

### 3.3. Molecular Docking: Visualizing the Interactions between PFSA Small Molecules and Large Proteins

Molecular docking, a computational simulation technique, enables rapid and clear visualization of the interactions between small ligand molecules and large protein molecules [29,30]. The molecular docking process was executed utilizing Autodock software 1.5.7 (Scripps Research, San Diego, CA, USA), targeting the interaction between the ligand small molecules and GPER. Of the 20 conformations obtained, the one possessing the lowest and most stable energy was selected for further analysis. Subsequently, VMD software 1.9.4a53 (NIH resource for macromolecular modeling & visualization, Champaign, IL, USA) [31] and LigPlot software 2.2.8 (EMBL’s European Bioinformatics Institute, Hinxton, Cambridgeshire, UK) [32] were employed to, respectively, visualize and interpret the results. In the visualization analysis of the interactions between residues and PFSAs small molecules, red areas denote strong interactions, green areas represent weak interactions, and gray areas suggest a tendency towards no interaction.

**Figure 3:**
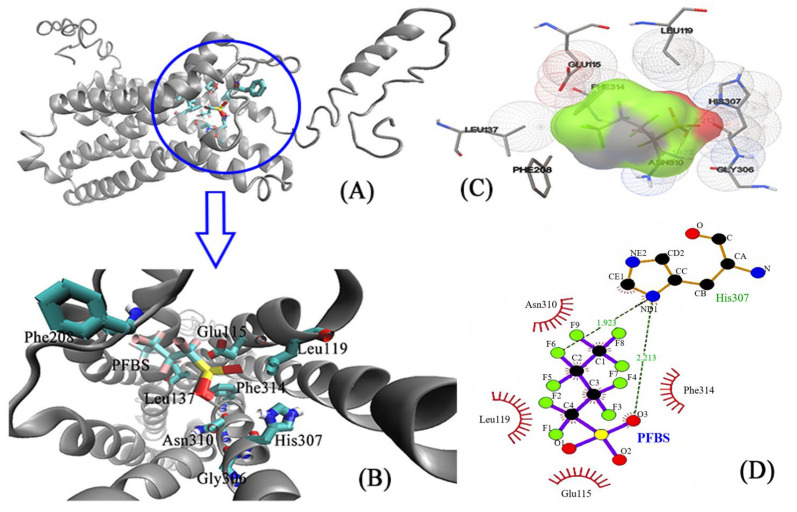
The molecular docking of PFBA and GPER. (**A**) Protein and the ligand. (**B**) The interacting amino acids on the binding site. (**C**) Different angles of amino acids at the binding site. (**D**) Strong interaction between the PFBS ligand and His307.

**Figure 4:**
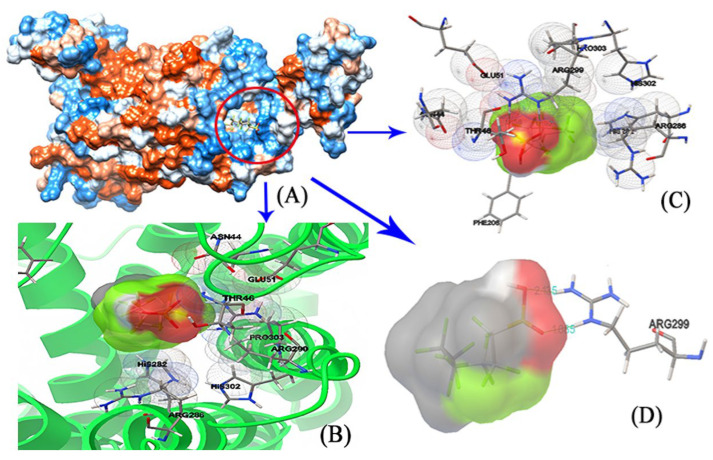
The molecular docking of PFHA and GPER. (**A**) The entire protein and the ligand. (**B**) The interacting amino acids on the binding site. (**C**) Different angles of amino acids at the binding site. (**D**) Stronger interaction between the ligand and Arg299.

The small molecule PFBS enters the protein’s cavity at the entrance and interacts with various amino acid residues in GPER, including Glu115, Leu119, Leu137, Phe208, Gly306, His307, Asn310, and others, as shown in Figure 3A,B. In addition, Figure 3C,D demonstrates that PFBS not only interacts with certain residues but also forms two hydrogen bonds with His307, with distances of 1.923 Å and 2.213 Å. The shorter distance of the hydrogen bonds indicates a stronger interaction force, which plays a crucial role in the stability of the complex. Furthermore, Figure 4 illustrates the molecular docking diagram between GPER and PFHS, in which PFHS docks at a position similar to PFBS, both located at the entrance cavity of the protein. The residues in the vicinity of PFHS primarily include Asn44, Thr46, Glu51, Phe208, His282, Arg286, Arg299, His302, Pro303, and others. Notably, the PFHS small molecule forms two robust hydrogen bonds with Arg299 at distances of 1.883 Å and 2.153 Å, significantly augmenting the stability of the entire complex system.

**Figure 5:**
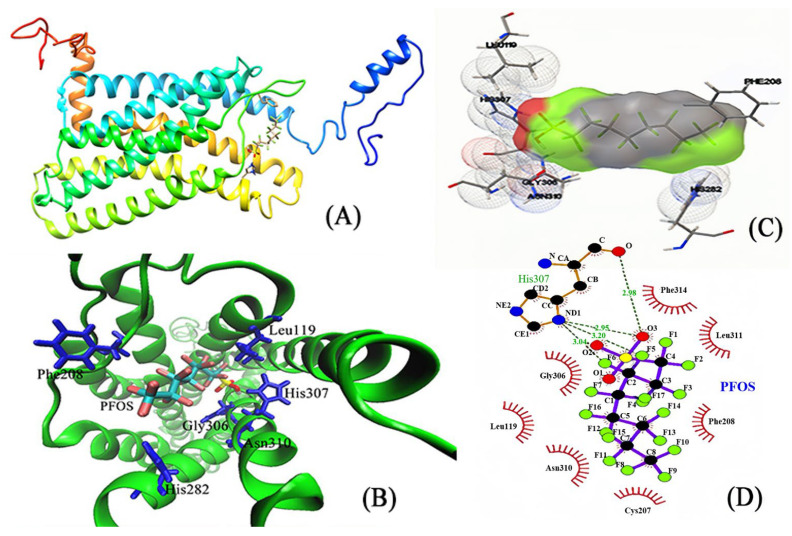
The molecular docking of PFOA and GPER. (**A**) Protein and the ligand. (**B**) The interacting amino acids on the binding site. (**C**) Different angles of amino acids at the binding site. (**D**) Strong interaction between the PFOS ligand and His307.

In the PFOA–GPER complex system, PFOA has the ability to enter the hydrophobic cavity located at the protein’s front, enabling it to exert its effect (Figure 5A). Figure 5B,C show the amino acid residues surrounding PFOS that interact with PFOS. Figure 5D illustrates hydrophobic interactions between PFOS and specific amino acid residues, such as Leu119, Cys207, Phe208, Gly306, His307, Asn310, Leu311, and Phe314. Furthermore, PFOS forms four hydrogen bonds with the His307 residue at distances of 2.59 Å, 2.98 Å, 3.04 Å, and 3.20 Å, which significantly contribute to the stability of the complex system.

**Figure 6:**
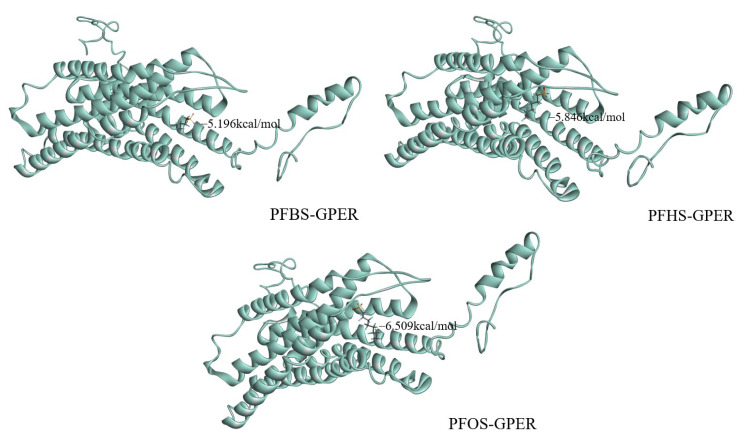
Binding energy of PFSAs to proteins.

The BE (binding energy) and LE (ligand efficiency) of proteins with ligands are key parameters in the fields of molecular docking experiments, providing vital information about the interactions between molecules. The BE denotes the intensity of the intermolecular interaction between a protein and a ligand, with a lower BE signifying a more stable interaction, thereby suggesting a tighter complexation of the ligand with the protein. As illustrated in Figure 6, the BE of PFBS, PFHS, and PHOS with GPER are −5.196 kcal/mol, −5.840 kcal/mol, and −6.509 kcal/mol, respectively. These relatively low BEs indicate a stable binding interaction between these PFSAs and GPER. The LE values of each PHSA for GPER calculated from the LE Equation (6) demonstrate that all PHSAs achieve good levels of LE against GPER; the LE of PFBS, PFHS, and PHOS with GPER are 0.306 kcal/mol/heavy atom, 0.256 kcal/mol/heavy atom, and 0.2246 kcal/mol/heavy atom, respectively, reflecting a relatively efficient ligand performance.

(6) $$LE={∆G}_{binding}÷N_{heavy}$$

In the formula, Δ*G_binding_* represents the binding free energy, which is typically expressed in units of kcal/mol, and *N_heavy_* refers to the number of non-hydrogen atoms in the ligand molecule.

Overall, the results obtained from molecular docking experiments demonstrate that PFSA and GPER can effectively dock with one another, and the primary interactions between PFSAs and GPER are driven by hydrophobic forces and hydrogen bonding. Moreover, the entry of PFSAs into the cavity of the GPER protein causes alterations to its native microenvironment, resulting in the quenching of GPER protein fluorescence. Therefore, the findings from the molecular docking experiments provide a reasonable explanation for the observed steady-state fluorescence experiments.

## 4. Secondary Structural Changes

### 4.1. Impact of PFS Binding on Protein Secondary Structure: Insights from Fourier Transform Infrared (FTIR) Spectroscopy

Fourier transform infrared (FTIR) spectroscopy is a widely employed technique for quantitatively analyzing protein secondary structure. Protein characteristic peaks are primarily observed within the range of 1600–1700 cm^−1^ [33,34]. More specifically, the following correspondences exist: 1610–1640 cm^−1^ for *β*-folds, 1640–1650 cm^−1^ for irregular coils, 1650–1660 cm^−1^ for *α*-helices, 1660–1680 cm^−1^ for *β*-turns, and 1680–1692 cm^−1^ for *β*-antiparallel structures [35]. Employing deconvolution fitting and integration methods subsequently enables the calculation of each component’s content in protein secondary structure.

**Figure 7:**
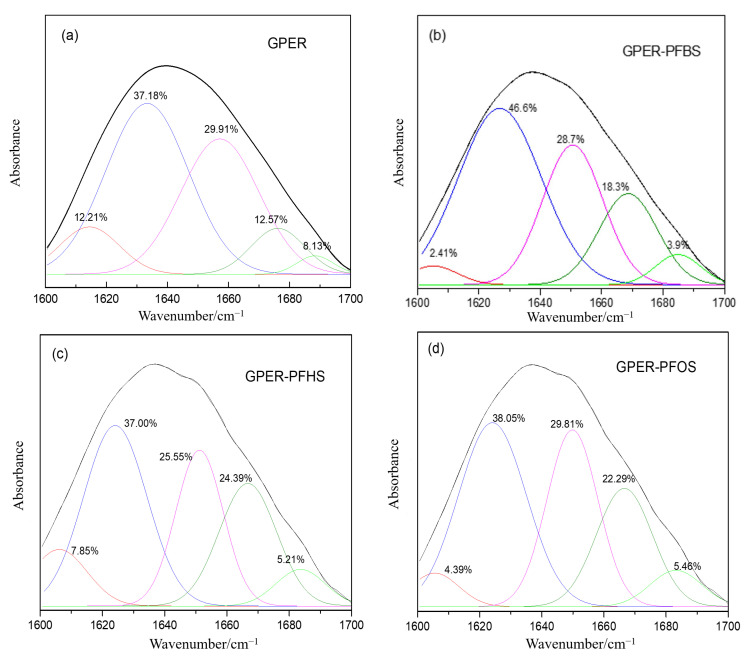
Fourier transform infrared spectroscopy of the percentage composition of secondary structures for free GPER and GPER–PFSA complex systems: free GPER (**a**), GPER–PFBS (**b**), GPER–PFHS (**c**), GPER–PFOS (**d**).

Figure 7 depicts the fitting of infrared spectroscopy data using deconvolution, resulting in a peak distribution chart for the system. Subsequently, employing integration methods allows for calculating the area under each individual peak, providing data in percentages. Compared to free GPER, the addition of PFBS, PFHS, and PFOS results in a decrease in *α*-helix content from 29.91% to 28.7%, 25.55%, and 29.81%, respectively. Additionally, the *β*-fold content of GPER decreases from 12.21% to 2.41%, 7.85%, and 4.39%, respectively. The *β*-antiparallel structures of GPER exhibit corresponding decreases, whereas the *β*-turn experiences a certain degree of increase. The irregular coil shows varying patterns. The experimental results indicate that the addition of pollutant small molecules (PFSAs) and their binding to GPER lead to modifications in the protein’s microenvironment. Consequently, alterations in the protein’s secondary structure occur, impacting normal physiological functions in the human body.

### 4.2. Validation of Protein Conformational Changes upon Small Molecular Ligand Binding Using Three-Dimensional Fluorescence Spectrum

Three-dimensional fluorescence spectroscopy has been widely employed to investigate conformational changes in protein macromolecules before and after binding with small molecular ligands [36,37,38]. To further validate the conclusions derived from the aforementioned Fourier transform infrared spectroscopy experiments, we analyzed the changes in GPER before and after binding to PFSA small molecules using three-dimensional fluorescence spectroscopy. The Rayleigh scattering peaks are represented by peaks where Em and Ex are equal. Peak a indicates changes in the helical, folding, and peptide chain structures of the protein, whereas peak b depicts the spectral behavior of fluorescent amino acid residues.

**Figure 8:**
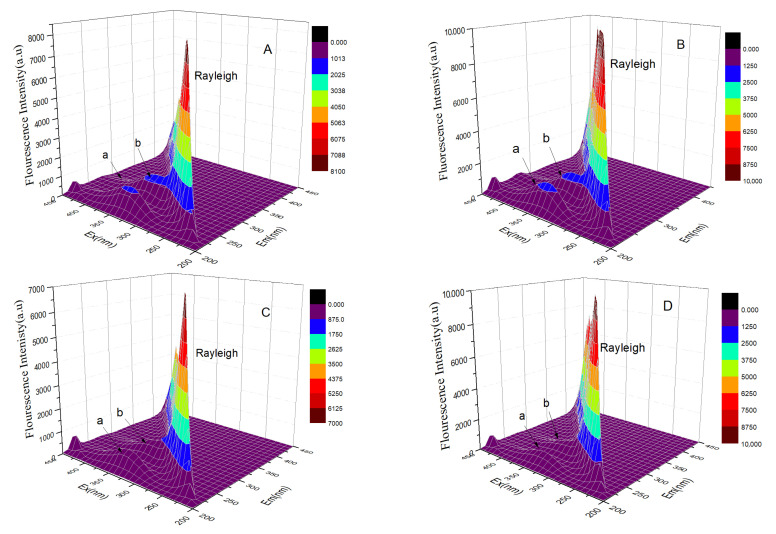
The three-dimensional fluorescence spectra of free GPER (**A**), GPER–PFBS (**B**), GPER–PFHS (**C**), and GPER–PFOS (**D**); Peak a: the changes in the helical, folding, and peptide chain structures of the protein, peak b: the spectral behavior of fluorescent amino acid residues.

Through a comparison of the free GPER with three other systems’ three-dimensional fluorescence, we observed an enhancement in the signals of peaks a and b upon the addition of PFBS, as depicted in Figure 8B. Additionally, the peak experiences a blue shift of approximately 3 nm, signifying that the addition of PFBS augments the hydrophobicity of the protein’s microenvironment, thereby mitigating collisions between fluorescent molecules and water molecules. However, with the addition of PFHS and PFOS (Figure 8C,D), the fluorescence intensity of both peaks decreases, accompanied by slight shifts in the positions of peaks a and b. This suggests that the addition of PFHS and PFOS modifies the microenvironment of GPER, resulting in modifications to the secondary structure of the protein as a whole. Simultaneously, the results obtained from three-dimensional fluorescence validate the accuracy of the Fourier transform infrared spectroscopy results.

## 5. Molecular Dynamics Simulation and Thermodynamic Analysis

### 5.1. Structural Stability and Ligand Binding Dynamics of GPER and GPER–PFSA Complexes Investigated through Molecular Dynamic Simulation

**Figure 9:**
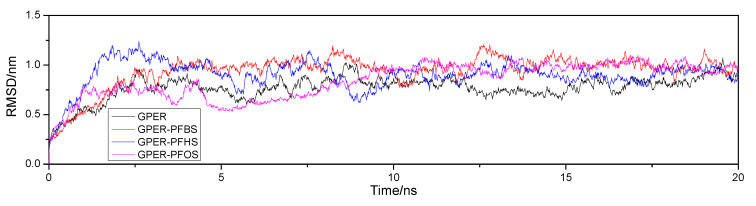
The RMSD of free GPER and GPER–PFSA complexes from 20 ns of MD simulations.

Performing a 20 nanosecond (ns) molecular dynamics simulation (MD) on the free GPER and GPER–PFSA complex systems using GROMACS 4.6.5 software. Extracting the root mean square deviation (RMSD) of the free GPER and GPER–PFSA complex systems after the simulation to analyze the stability of their structures. Figure 9 illustrates the RMSD values for each system using plotted lines. The graph shows significant fluctuations in the data during the initial 10 ns. Subsequently, between the 10 to 15 ns timeframe, the data start to stabilize, leading to a largely stable equilibrium state by the 15 to 20 ns interval. This variability indicates the overall stability of the system. By calculating the variance of the data within the 15 ns to 20 ns timeframe, the variances are determined as follows: 0.0657 (±0.0005) nm for free GPER, 0.0514 (±0.0005) nm for GPER–PFBS, 0.0572 (±0.0005) nm for GPER–PFHS, and 0.0413 (±0.0005) nm for GPER–PFOS. It is noteworthy that the variance of the free GPER is the highest, suggesting that the stability of the generated complexes exceeds that of the free GPER.

**Figure 10:**
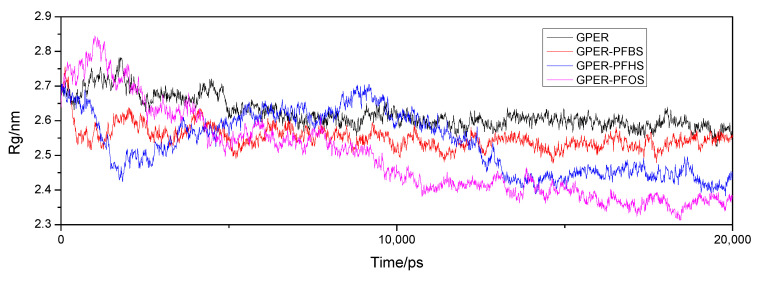
The Rg of free GPER and GPER–PFSA complexes from 20 ns of MD simulations.

**Figure 11:**
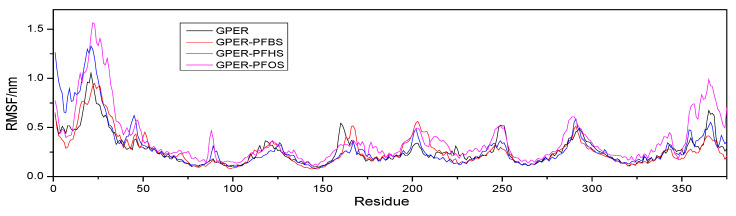
The root mean square fluctuation of the free GPER and GPER–PFSA complex systems during the 20 ns MD simulation.

The radius of gyration (Rg) can be used to evaluate the molecular backbone structure of the free GPER and GPER–PFSA complex systems [39]. During the initial 5 ns, both the free protein and the GPER–PFSA complex exhibit significant fluctuations, as shown in Figure 10. From 5 to 15 ns, relatively stable variations are observed. In the final 5 ns, all four systems reach a gradual plateau, indicating a tendency towards stability. Upon observation, it can be noticed that the free GPER has the largest Rg value, which gradually decreases with an increase in the number of carbon atoms in the ligand small molecules within the system. Averaging the data from 15 ns to 20 ns, the results indicate that the free GPER is approximately 2.5872 (±0.005) nm, the GPER–PFBS system is around 2.5371 (±0.0005) nm, the GPER–PFHS complex system is approximately 2.4431 (±0.0005) nm, and the GPER–PFOS system is approximately 2.3674 (±0.0005) nm. The results suggest that incorporating small PFSA ligand molecules leads to a contraction in the GPER structure. Moreover, as the number of carbon atoms increases, the magnitude of this contraction tends to increase as well. Additionally, the binding of these small molecules to GPER simultaneously alters the protein’s microenvironment, leading to changes in the secondary structure. The simulation outcomes corroborate Fourier transform infrared spectroscopy and three-dimensional fluorescence results. MD simulations enable the examination of the local variations in protein residues by analyzing root mean square fluctuation (RMSF) [40]. The simulation results depicted in Figure 11 illustrate that both the free GPER and the GPER–PFSA complex system exhibit similar fluctuation trends throughout the entire process. However, the fluctuation observed in GPER–PFOS appears to be relatively more significant compared to the other systems, indicating a greater impact of PFOS on the residues and microenvironment of GPER. Furthermore, the free GPER consistently displays anomalous fluctuations within residues 180–220, with lower RMSF values compared to the GPER–PFSA complex system. This observation, coupled with molecular docking simulations, suggests a probable binding of the small-molecule ligand in this region.

**Figure 12:**
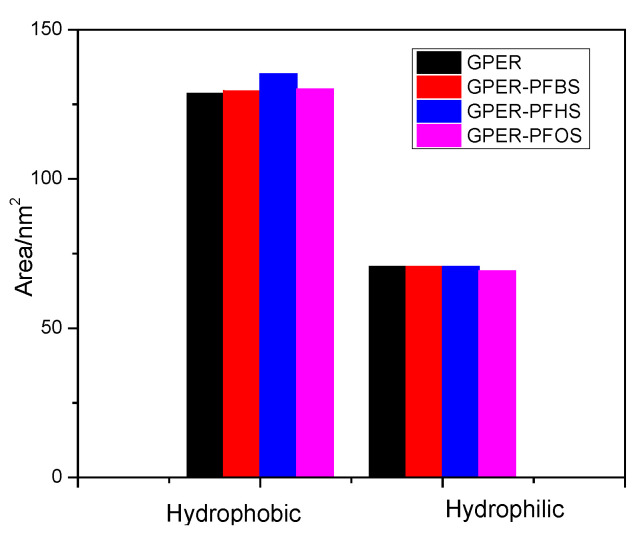
The average solvent-accessible surface area of free GPER and GPER–PFCA complexes.

The g_sas module in the GROMACS program was utilized to analyze the solvent-accessible surface area of the free GPER and GPER–PFSA complex systems. Subsequently, the hydrophobicity and hydrophilicity of GPER were determined before and after binding small ligand molecules. The obtained results, as depicted in Figure 12, indicate an increase in hydrophobicity across the three types of GPER–PFSA complexes compared to the free GPER. Among these complexes, PFBS and PFOS systems show relatively minor increases, whereas the PFHS molecule exhibits the most significant rise in hydrophobicity. Meanwhile, the hydrophilicity of each system remains relatively consistent, with an acceptable margin of error. The solvent-accessible surface area results indicate the entry of the small molecules into the GPER cavity, resulting in alterations in the microenvironment and secondary structure of GPER.

### 5.2. Thermodynamic Analysis of Protein–Small Molecule Ligand Interactions: Significance of Hydrophobic Interactions in the Binding of PFSAs with GPER

In an aqueous solution, the interactions between proteins and small-molecule ligands mainly involve hydrogen bonding, hydrophobic interactions, electrostatic forces, and van der Waals forces. The *ΔH* and *ΔS* obtained through the MM–PBSA (Molecular Mechanics/Poisson–Boltzmann Surface Area) method can be used to discern the type of interaction between the protein and ligand. According to the literature records [41,42], positive values for both ∆*H* and ∆*S* indicate hydrophobic forces, negative values indicate hydrogen bonds and van der Waals interactions, and when ∆*H* < 0 and ∆*S* > 0, it implies electrostatic attraction. The calculated values for ∆*G*, ∆*H*, and ∆*S* of each system using thermodynamic Formulas (7)–(9) [43,44,45] are presented in Table 1. Upon analysis of the computed data, it is evident that ∆*G* is consistently negative, while ∆*S* and ∆*H* are always positive during the binding process of PFSAs with GPER. This indicates that the binding process of GPER with the small-molecule ligand is spontaneous. According to the aforementioned principles, hydrophobic interactions serve as the primary force driving the binding between PFSA and GPER.

(7) $$ln{(K}_{2}/K_{1})=∆H\left(1/T_{1}-1/T_{2}\right)/R$$

(8) $$∆G=-RTlnK_{a}\;$$

(9) ∆G=∆H−T∆S 

## 6. Conclusions

This article combines various spectroscopic methods with molecular docking, molecular dynamics simulations, and other simulation techniques to assess, at a molecular level, the interaction mechanism between the pollutant small molecule PFSAs and GPER. Steady-state fluorescence spectra indicate that PFSAs can bind to the cavity of the GPER protein, leading to fluorescence quenching. Analysis of the data suggests that the mechanism of fluorescence quenching is static quenching. Analysis of the overlapped UV–visible and fluorescence spectra reveals that the distance between the small molecule PFSAs and the fluorescent amino acids of GPER is less than 7 nm. This suggests the presence of non-radiative energy transfer between GPER and PFSAs. Fourier transform infrared spectroscopy and three-dimensional fluorescence results indicate that the small molecule enters the hydrophobic cavity of GPER, causing changes in the protein’s native microenvironment and consequently leading to alterations in the protein’s secondary structure. The results of molecular dynamics simulations indicate that the stability of the GPER–PFSA complex system surpasses that of the free GPER. Moreover, with the formation of the complex, the protein’s radius of gyration (Rg) decreases, signifying a contraction in the protein’s structure. This further validates the accuracy of Fourier transform infrared spectroscopy and three-dimensional fluorescence results. The results obtained from the thermodynamic analysis suggest that the predominant interaction between PFSAs and GPER is through hydrophobic forces, aligning well with the molecular docking outcomes. In conclusion, the pollutant small molecule PFSAs can bind to GPER, forming a stable complex, consequently altering some inherent properties of GPER and potentially affecting normal physiological functions in living organisms. Researching the interactions between protein macromolecules and pollutant small molecules has gained substantial traction. Innovative approaches continually emerge, propelling research in the field of interactions and significantly contributing to understanding the absorption, distribution, transportation, and metabolism of pollutant small molecules within the human body. Furthermore, this research aids in elucidating the combined toxicological effects of various pollutant small molecules and facilitates the development of detoxifying agents, providing vital theoretical foundations and reference values. Therefore, this study holds profound significance in environmental science, toxicology, chemistry, life sciences, and related fields in advancing knowledge and application.

## Figures and Tables

**Table 1:** The thermodynamic parameters of PFSA and GPER interactions at different temperatures.

	T/K	∆*H*/(kJ·mol)	∆*G*/(kJ·mol)	∆*S*/(J·mol·K^−1^)
PFBS	293 K	21.95	−33.75	190.11
	298 K		−34.74	
	303 K		−35.65	
PFHS	293 K	8.83	−34.22	146.89
	298 K		−34.92	
	303 K		−35.69	
PFOA	293 K	49.99	−34.20	287.18
	298 K		−35.50	
	303 K		−37.07	

## Data Availability

The data presented in this study are available on request from the corresponding author.

## Appendix A

**Table A1:** The quenching constant, quenching rate constant, apparent binding constant, and number of binding sites of PFSA–GPER interactions at different temperatures.

	T/K	*K_sv_*	*K_q_*	*K_a_*	n
PFBS	293 K	1.47 × 10^5^	1.47 × 10^13^	1.04 × 10^6^	0.9825
	298 K	1.90 × 10^5^	1.90 × 10^13^	1.23 × 10^6^	0.9735
	303 K	2.31 × 10^5^	2.31 × 10^13^	1.40 × 10^6^	0.9670
PFHS	293 K	1.89 × 10^5^	1.89 × 10^13^	1.26 × 10^6^	0.9786
	298 K	2.19 × 10^5^	2.19 × 10^13^	1.32 × 10^6^	0.9695
	303 K	2.58 × 10^5^	2.58 × 10^13^	1.42 × 10^6^	0.9693
PFOS	293 K	1.41 × 10^5^	1.41 × 10^13^	1.25 × 10^6^	0.9961
	298 K	1.69 × 10^5^	1.69 × 10^13^	1.67 × 10^6^	0.9943
	303 K	2.35 × 10^5^	2.35 × 10^13^	2.46 × 10^6^	0.9986
